# Are failures to look, to represent, or to learn associated with change blindness during screen-capture video learning?

**DOI:** 10.1186/s41235-018-0142-3

**Published:** 2018-12-27

**Authors:** Daniel T. Levin, Adriane E. Seiffert, Sun-Joo Cho, Kelly E. Carter

**Affiliations:** Department of Psychology and Human Development, Vanderbilt University, Peabody College #552, 230 Appleton Place, Nashville, TN 37203-5721 USA

## Abstract

Although phenomena such as change blindness and inattentional blindness are robust, it is not entirely clear how these failures of visual awareness are related to failures to attend to visual information, to represent it, and to ultimately learn in visual environments. On some views, failures of visual awareness such as change blindness underestimate the true extent of otherwise rich visual representations. This might occur if people did represent the changing features but failed to compare them across views. In contrast, other approaches emphasize visual representations that are created only when they are functional. On this view, change blindness may be associated with poor representations of the changing properties. It is possible to compromise and propose that representational richness varies across contexts, but then it becomes important to detail relationships among attention, awareness, and learning in specific, but applicable, settings. We therefore assessed these relationships in an important visual setting: screen-captured instructional videos. In two experiments, we tested the degree to which attention (as measured by gaze) predicts change detection, and whether change detection is associated with visual representations and content learning. We observed that attention sometimes predicted change detection, and that change detection was associated with representations of attended objects. However, there was no relationship between change detection and learning.

**Electronic supplementary material:**

The online version of this article (10.1186/s41235-018-0142-3) contains supplementary material, which is available to authorized users.

## Significance

Much research has demonstrated that people fail to detect visual changes, and some of this work has demonstrated that these failures can occur in important applied settings. In any given setting, however, it is not clear what the implications of these failures are. For example, when someone misses a visual change (such as the substitution of one icon for another) when learning about a graphical computer interface, did this failure occur simply because they failed to look at the changing properties, or did the learner look at the changing properties but fail to create a representation sufficiently durable to see the change? Moreover, when someone misses a change, how broad was their failure? Does a failure to see changes imply a failure to remember the visual features of the interface or, even more broadly, a failure to pay much attention at all to the contents of the lesson? We therefore assessed change blindness in a specific real-world setting: screen-captured instructional videos. We tested the degree to which change blindness in this setting occurs because of failures to look at the changing properties and whether these visual-cognitive failures are associated with failures to remember visual properties and failures to learn instructional contents.

### Attention, awareness, and learning in a naturalistic setting

Despite the clear importance of maintaining effective visual awareness, research exploring phenomena such as change blindness and inattentional blindness reveals how people often fail to detect visual changes and unexpected objects in their environments (Jensen, Yao, Street, & Simons, 2011; Levin & Baker, 2015; Simons & Levin, 1997; Varakin, Levin, & Fidler, 2004). These failures of awareness imply that viewers are not representing and remembering what they see, but this seems to conflict with research that documents people’s ability to represent large amounts of visual information and more generally to learn from their experiences. One means of reconciling this apparent conflict is by assuming that change blindness is a very narrow failure that occurs in the face of otherwise effective visual representations. For example, imagine that someone watching an informational video teaching spreadsheet formulas fails to notice that a menu icon suddenly changes color. This could be a sign that they have failed to represent anything about the icons and by extension have not learned much at all about the videos. However, it is also possible that the viewer remembers plenty about the icons—they just didn’t *compare* the pre- and post-change views, and more generally, their failure to detect the change may say little about the degree to which they have learned the contents of the video.

As we will review below, there is evidence for both of these views. So, some situations produce evidence that change blindness is associated with failures to represent visual properties, while in other settings change blindness seems to occur in spite of otherwise-accessible representations of the changing properties. This makes it important to ask whether visual failures in specific settings are broad or narrow. So, if change blindness is caused by a broad representational failure, it can be used both as a sign that viewers will fail to learn important things (such as the identity of specific icons in the above example) and as a target of possible intervention to improve task performance by highlighting important changes. In this paper we describe two experiments testing change detection in a visual learning setting: screen-captured instructional videos. Our primary question was whether change blindness would reflect failures to represent the changing properties themselves or whether change blindness was caused by a failure to compare otherwise effectively represented properties. We also assessed eye movements to test whether change detection occurs in the face of gaze at both pre- and post-change objects, and whether increased gaze would be associated with increased change detection. Finally, we assessed learning to test whether change blindness would be a sign of a global failure to learn from the videos.

#### Representation and comparison failures in naturalistic dynamic settings

One might conclude that change detection failures are evidence that merely looking at a scene does not automatically generate extensive visual representations of the things in that scene unless additional processes are invoked (Chen, Swan, & Wyble, 2016; Levin & Baker, 2015; Rensink, 2000). A number of findings support this representation-failure hypothesis. For example, Caplovitz, Fendrich, and Hughes (2008) coined the term “attentive blank stares” to describe the relatively frequent cases of gaze falling upon both pre- and post-change properties in the absence of change detection (see also Fudali-Czyż, Francuz, & Augustynowicz, 2014). Change detection failures in the face of verified looking have been observed in realistic settings such as slight-of-hand magic tricks (O’Regan, Deubel, Clark, & Rensink, 2000; Smith, Lamont, & Henderson, 2012).

Other research has relied upon behavioral paradigms to assess the degree to which change blindness is associated with failures to represent pre- and post-change objects. Levin, Simons, Angelone, and Chabris (2002) found a strong relationship between change detection and subsequent visual recognition of the changing objects. In these experiments, the participants interacted directly with an experimenter who was surreptitiously replaced with another person during the interaction. After reporting whether they saw the change, participants were asked to recognize the experimenter from a forced-choice lineup. A substantial proportion of participants missed the change, and participants who missed the change were often at chance when attempting to recognize both the pre- and post-change experimenters, while participants who reported the change did considerably better. Similar links between change detection and recognition have been observed in participants who viewed videos depicting crimes in which one actor was substituted for another (Davies & Hine, 2007; Nelson et al., 2011). It is even possible to argue that these representational failures may ultimately result in failures to learn about repeatedly presented visual information both in the lab (Wolfe, Klempen, & Dahlen, 2000) and in our everyday visual environment (see, for example, Nickerson, 1965; Nickerson & Adams, 1979).

However, considerable evidence also supports the hypothesis that change blindness can occur even if viewers have represented the pre- and post-change objects but have failed to compare those representations (Scott-Brown, Baker, & Orbach, 2000; Simons, 2000). Also, although fixation is no guarantee that change detection will occur, several studies have demonstrated that change detection is sometimes more likely with fixation (Hollingworth, Schrock, & Henderson, 2001; Hollingworth, Williams, & Henderson, 2001). As many commentators have pointed out, viewers are often able to represent a large amount of visual information with comparatively little effort or control (Olson, Moore, & Drowos, 2008). Classic research demonstrates good picture recognition memory, even for thousands of pictures (Konkle, Brady, Alvarez, & Oliva, 2010; Nickerson, 1965; Shepard, 1967; Standing, 1973). Other work exploring visual statistical learning supports a similar hypothesis by demonstrating that participants learn relationships between a large number of targets and their contexts, or sequential contingencies among serially presented objects (Turk-Browne, Jungé, & Scholl, 2005).

In addition, more naturalistic experiments do sometimes demonstrate that change blindness can be associated with fully effective representations of pre- and post-change objects. For example, Angelone, Levin, and Simons (2003) asked participants to watch a short video showing people conversing. Changes to object included color changes in clothing, or the identity of one of the actors in the video. Not only did participants who missed the change recognize the changing properties at above-chance levels, but they were just as accurate at recognizing the properties as participants who saw the changes. These results dissociate change detection and recognition, suggesting that change blindness may in some cases underestimate the extent of visual representations. Other research demonstrates that people can identify previously seen objects in detail (Hollingworth & Henderson, 2002; Hollingworth, Williams, & Henderson, 2001), even when the recognition test is not expected (Castelhano & Henderson, 2005; Varakin & Levin, 2006; Williams, Henderson, & Zacks, 2005).

In the face of evidence like this, it is important to consider the possibility that participants in real-world change detection experiments are inattentive or overly focused on a social interaction. Thus, poor visual recognition among change-missers may represent an exceptionally low ebb of visual processing (Beck & Levin, 2003; Landman, Spekreijse, & Lamme, 2003). Therefore, it seems critical to explore both the prevalence of change blindness and the relationship between change blindness and visual representation in a variety of settings that characterize important real-world processing. It is especially useful to explore a setting characterized by a rich conceptual framework that participants are motivated to learn from. Participants in this study watched screen-captured instructional videos designed to teach them how to perform specific tasks, then were tested on their memory for the contents of the videos. At some point during the videos, a visual change occurred (for example, a colored region on a banner graphic changed from green to blue; see Additional file 1 for illustrations of all changes), and participants were forewarned of this possibility. Thus, in this setting, participants were both motivated to attend to visual information and aware that they would be asked to detect visual changes.

We asked two basic questions. First, how broad is the representational failure associated with change blindness in a real-world learning setting? We tested whether participants who missed changes also had difficulty recognizing the changing properties, and whether these missers learned less effectively from the videos. Second, we tracked participants’ gaze while viewing the videos. If change blindness is caused by a failure to look at the changing objects, then it should rarely occur when gaze has fallen upon both the pre- and post-change objects, and increased looking at the changing objects should be associated with increased change detection and more accurate visual recognition.

## Experiment 1

### Method

#### Participants

Eighty Vanderbilt University undergraduates (mean age = 18.8 years, range 18 to 22, 58 female) completed experiment 1 in exchange for course credit.

#### Stimuli

Participants viewed four brief screen-captured instructional videos, two describing how to use Google Forms to create and distribute a simple survey (3 min 56 s and 5 min 38 s in duration), and two describing how to create charts in Google Sheets (4 min 09 s and 6 min 27 s in duration). Each video consisted of a replayed interaction with a computer application accompanied by narration from the individual interacting with the application. Both pairs of videos delivered related lessons, with the second member of each pair building upon the lesson in the first member of each pair. The videos were intentionally created to include a range of central and noncentral actions and information, and so were relatively unrehearsed, and included several errors and instances of backtracking.

Each video included a visual change occurring at an unexpected time. In the first Google Forms video a set of three icons in the middle of the screen changed into other icons (at 3 min 11 s from the beginning of the video; see Additional file 1 for illustrations of all changes), and in the second a medium-sized colored region in a graphic changed color (at 4 min 13 s). In the first Google Sheets video, the change was in a colored region at the upper left of the screen (at 1 min 10 s). In the second Google Sheets video, the change was a color change to a set of graphics in a dialog box in addition to a change to the size of the elements due to a video rendering error (at 1 min 4 s). In all cases, the changes were intended to be near the current center of interest in the videos, but not within it. Each change was accompanied by a brief blank screen (three frames, 100 ms) and a seven-frame beep (beginning two frames before the blank and ending two frames after). All property changes were counterbalanced such that one group of participants saw an A to B change and the other saw the B to A version of the change. All stimuli are available at https://osf.io/fgpdc/.

#### Procedure

Before viewing the videos, participants were told that they would be tested on those contents. Participants were also informed that there would be a visual change during the video accompanied by a brief tone, and that they would be asked whether they detected the change. Participants were then calibrated in the eye tracking system (a desk-mounted Eyelink 1000 eye tracker with a chin rest) then viewed the four videos. Videos were viewed on a 27-inch monitor (screen size 51.4 cm × 32.4 cm) from a viewing distance of approximately 56 cm. After each video, participants were first asked whether they had detected the visual change that had occurred in the video, and if so, to describe what had changed. Then, participants completed a two-alternative forced choice recognition test for the pre-change object in which they viewed two different versions of the pre-change object and were asked to choose which they thought was present before the change. Finally, they completed another recognition test for the post-change object. After viewing all of the videos, participants were asked to complete a multiple choice content post-test to assess learning from the videos. Gaze was recorded at 1000 Hz, but for analysis was re-binned to 30 Hz (one sample per video frame) by averaging of x- and y-coordinates.

In addition to testing change detection, these experiments were part of a larger project exploring the effects of instructional context on links between eye movements and learning. Therefore, half of the participants in experiment 1 viewed the videos having been instructed to focus on the causal structure of the information presented while the other group of participants were instructed to attend to the sequence of actions. To reinforce these instructions, participants were asked either two causal or two sequence questions after each video. This manipulation was uniform in producing no impact in either experiment reported here, and the questions were not part of the pre- or post-tests reported below, so this manipulation will not be discussed further.

#### Data coding

Because participants sometimes report events as changes that were not the changes we were interested in, change detection responses were coded as hits only if participants reported seeing the change and described something that could plausibly be the changing property. In cases where the description was ambiguous (for example, “an object on the screen changed”), responses were coded as change detections. However, in cases where participants reported noticing change but then described something that was identifiably not the changing object (for example, “I saw the screen flash” or “the screen got bigger”), the responses were coded change-misses.

### Results

#### Change detection and recognition of changing properties

**Fig. 1:**
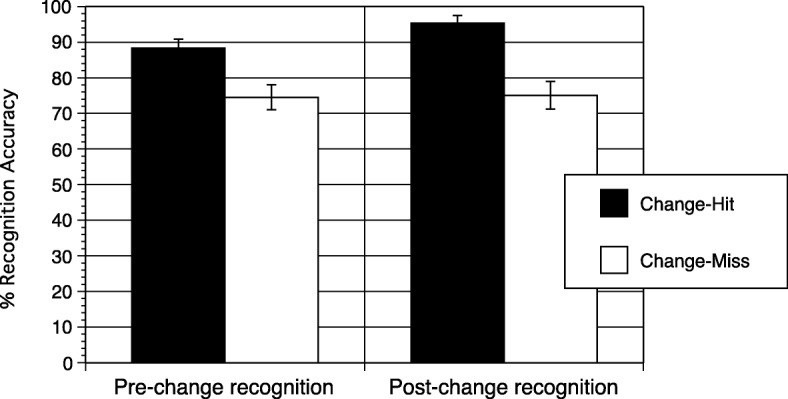
Percent accuracy on visual recognition for change-hit and change-miss trials. *Error bars* represent standard errors

Overall, participants detected 57% of changes across the four videos. Visual recognition of changing properties was more accurate for trials in which participants detected changes (92%) than on trials in which participants missed changes (75%; *t*(66) = 4.421, *p* < 0.001, *d* = 0.540). Recognition was more accurate on change-hit trials than on change-miss trials both for pre-change properties (88% vs 75%; *t*(66) = 2.594, *p* = 0.012, *d* = 0.317) and for post-change properties (95% vs 75%, *t*(66) = 4.091, *p* < 0.001, *d* = 0.500; Fig. 1). Note that because this analysis was a paired *t*-test comparing recognition accuracy between change-hits and change-misses within subjects, some participants were dropped from the analysis if none of their four video-viewings resulted in a hit or a miss. Therefore, these effects were confirmed with mixed-effects logistic regressions across the four videos (with subjects as the group variable). This analysis confirmed that detecting a change was associated with both improved pre-change recognition (odds ratio = 2.67, SE = 0.84, *z* = 3.13, *p* = 0.002) and improved post-change recognition (odds ratio = 11.73, SE = 6.86, *z* = 4.21, *p* < 0.001).

#### Gaze and change detection

To test for a link between gaze and change detection, we tested whether duration of gaze at pre- and post-change properties predicted change detection. To test this hypothesis we defined rectangular regions of interest bounding the pre-change and post-change properties and tabulated the number of frames (temporally contiguous or not) for which gaze fell within those regions. In video 2, the changing property was visible for the entire duration of the video, so we limited the assessed fixation durations to the 10 s before and after the change. These gaze durations were entered as predictors into a generalized estimating equations analysis with video, pre-change gaze duration, and post-change gaze duration predicting change detection. Increases in post-change (*X*^*2*^(1) = 11.580, *p* = 0.001) but not pre-change (*X*^*2*^(1) = 1.246, *p* = 0.264) gaze duration significantly predicted increased change detection.

We also assessed the relationship between gaze duration and change detection within each of the four videos using unpaired *t*-tests. As can be seen in Table 1, pre-change gaze duration significantly predicted change detection for none of the individual videos, while post-change gaze duration did significantly predict change detection for three of the four videos.

**Table 1:** Relationship between gaze duration in pre- and post-change ROIs and change detection for each video

Experiment 1		
	Frames in pre-change	Frames in pre-change
	ROI; change HIT (n)	ROI; change MISS (n)
Video 1	16.7 (13)	12.6 (65)
Video 2	9.8 (44)	14.9 (34)
Video 3	8.2 (41)	6.5 (37)
Video 4	39.2 (50)	35.4 (28)
	Frames in post-change	Frames in post-change
	ROI; change HIT	ROI; change MISS
Video 1	44.1 (13)	13.2 (65)***
Video 2	15.8 (44)	23.4 (34)
Video 3	34.3 (41)	14.0 (37)***
Video 4	232.6 (50)	167.5 (28)**
Experiment 2		
	Frames in pre-change	Frames in pre-change
	ROI; change HIT (n)	ROI; change MISS (n)
Video 1	7.6 (25)	7.5 (55)
Video 2	87.5 (2)	213.2 (78)
Video 3	5.1 (46)	4.7 (34)
Video 4	29.8 (4)	21.1 (76)
	Frames in post-change	Frames in post-change
	ROI; change HIT	ROI; change MISS
Video 1	14.2 (25)	9.0 (55)
Video 2	283.7 (2)	258.5 (78)
Video 3	4.7 (46)	3.4 (34)
Video 4	43.8 (4)	16.7 (76)

**p < 0.01, ***p < 0.001 unpaired *t*-tests

To test the relationship between gaze and representation we tested whether fixation on pre- and post-change properties would predict performance on the subsequent recognition test for pre- and post-change properties. In an initial analysis across all four videos, we used a general estimating equation procedure to test whether gaze duration in pre- and post-change regions of interest (ROIs) significantly predicted recognition of pre- and post-change properties (using video as a within-subject effect, assuming a binomial distribution, and an unstructured working correlation matrix structure). We ran two analyses, one with both pre- and post-change gaze duration predicting pre-change recognition, and one predicting post-change recognition. Results indicated that gaze durations predicted neither pre-change recognition (*p* values > 0.22), nor post-change recognition (*p* values > 0.14).

To assess the extent to which change blindness can occur in the face of gaze at both the pre- and post-change object, we tabulated the joint minimum number of frames that each participants’ gaze was in the pre- or post-change ROI for a given trial. So, if a given participant’s gaze fell within the pre-change ROI for 10 frames and within the post-change ROI for 15 frames, their 10-frame minimum would reflect the fact that they had at least 333 ms gaze within both regions to provide opportunity to encode the pre- and post-change properties. We also assessed the longest joint minimum continuous run length between pre- and post-change ROIs to assess the degree to which change blindness may have occurred in the face of relatively long continuous samples of visual information within both ROIs (that is, we calculated the longest temporally continuous run of frames that gaze fell in the pre-change ROI and in the post-change ROI and reported the lesser of these values).

We tested the degree to which minimum gaze duration predicted change detection across all four videos using a general estimating equations procedure (again using video as a within-subject effect, assuming a binomial distribution, and an unstructured working correlation matrix structure), and found that minimum gaze duration was not significantly different for detected changes (18.4 frames) vs nondetected changes (12.9 frames; *X*^*2*^(1) < 1). We also tested whether the joint minimum continuous run length between pre- and post-change ROIs would be different for detected and undetected changes. This analysis similarly revealed no significant difference between detected changes (8.59 frames) and undetected changes (7.35 frames; *X*^*2*^(1) < 1).

To illustrate the degree to which failures to detect changes were associated with gaze falling in both the pre- and post-change regions, we tabulated the percentage of missed and detected changes for which gaze durations in both the pre- and post-change regions afforded opportunity to detect the changes. For 60% of missed changes, gaze fell within both regions for at least five frames (167 ms). This is similar to Fudali-Czyż et al.’s (2014) 150-ms criterion for “attentive blank stares”. For 40% of missed changes, the gaze minimum was at least 10 frames and for 12% it was at least 30. This compares with 56% of detected changes for which gaze was in both regions for at least five frames, 34% of detected changes for which gaze was in both ROIs for more than 333 ms, and 2.7% of detected changes for which gaze was in both ROIs for at least 1 s. Similarly, for minimum longest within-ROI run-lengths, for 44% of missed changes gaze was in both regions for at least five continuous frames. For 23% of missed changes gaze was in both regions continuously for 10 frames, and for 3% it was continuously in both regions for 30 frames. In comparison, for 51% of detected changes, gaze was in both regions for at least five frames. For 31% of detected changes gaze was in both regions continuously for 10 frames, and for 3% it was continuously in both regions for 30 frames.

#### Change detection and learning

Overall, post-test scores demonstrated moderate understanding of the material. The mean score was 61%, 77%, 68%, and 73% correct for videos 1–4, respectively (compared with a nominal 25% guessing baseline for the four-alternative multiple choice questions used here). A regression predicting content test scores with change detection and recognition did not reveal significant predictors (*t* values < 1). Individual regressions predicting accuracy on causal items (test items that assessed conceptual knowledge of the lessons), sequence items (testing recognition of specific sequences of instructional steps), menu items (items testing visual recognition of the specific order and contents of drop-down menus), and detail questions (items assessing memory for small peripheral details) in no case included any significant predictors (*t* values − 1.578 and less).

### Discussion

Experiment 1 demonstrated that change blindness was prevalent while viewing screen-captured instructional videos, and that these failures were associated with less accurate recognition of the changing objects. This was true both for recognition of pre- and post-change object properties. That said, recognition was reasonably accurate and well above a nominal chance level even where changes were missed. In addition, the experiment demonstrated that visual processing, as measured by gaze, was associated with change detection, but this relationship was strongest for post-change visual properties. It is important to note that this relationship is fundamentally ambiguous because of the reasonable likelihood that participants would lengthen their gaze to inspect the post-change property. More telling is the finding that attentive blank stares appeared to be frequent, as participants often missed changes despite looking at both the pre- and post-change ROIs. Finally, there were no links between awareness/representation and content learning.

## Experiment 2

Although experiment 1 revealed a clear relationship between change detection and recognition of both pre- and post-change objects, and little relationship between either of these measures and learning, it is important to consider the possibility that both of these findings were idiosyncratic. In particular, the videos used in experiment 1 included a large number of peripheral actions. This was intentional, but it may have reduced participants’ willingness to represent and track visual features during the videos. Also, we included no pre-test in experiment 1, so post-test scores could have reflected existing knowledge as opposed to learning from the videos. To correct these issues, and to replicate our basic finding linking change detection with recognition, we ran another experiment, this time using videos designed to focus more on central information. The new videos were rehearsed and edited to include very little non-central information, and they also included very few verbal lapses or disfluencies. In addition, experiment 2 included a content-knowledge pre-test in the form of one open-ended content question for each video. Post-test questions were again multiple-choice questions.

### Method

#### Participants

Eighty-four participants completed the experiment. Of these, four were dropped due to eye tracking failures, leaving a total of 80 participants in the analysis (mean age = 22 years, 26 male). Participants were Vanderbilt University students who completed the experiment in exchange for course credit and members of the local community who completed it in exchange for $10 payment.

#### Stimuli

Participants viewed four instructional screen-captured videos, similar to those used in experiment 1. These videos were more practiced and included fewer noncentral actions. Two of the videos taught spreadsheet concepts in the context of Google Sheets (absolute vs relative reference and use of the sumproduct function), and two lessons described basic editing and compositing functions in the movie-editing application HitFilm. Changes were similar to those in experiment 1 (Additional file 1). Three of the changes were changes to the color of an on-screen object, and one was a change to the typeface name and size. All stimuli are available at: https://osf.io/fgpdc/.

#### Procedure

Procedures were similar to those in experiment 1. The only difference was that before participants were calibrated in the eye tracker, they completed a knowledge pre-test consisting of four open-ended questions The pre-test tested one concept from each video by asking participants to describe or define it. For example, the question for the first spreadsheet video asked participants to “Describe the difference between absolute and relative reference in a spreadsheet program such as Google Sheets or Microsoft Excel.”. Responses were an average of 11.09 words long, and each response was scored by two raters on a 0- to 3-point scale. The mean response across the two raters was used in the analysis, and the correlation between raters’ scores across all items was *r* = 0.77.

At the end of the experiment, participants were asked to rate the proportion of their effort (0 to 100%) they devoted to the change detection task. This addition was made after the first 19 participants were run, so analyses including this measure included only 61 participants.

### Results

#### Change detection and recognition of changing properties

**Fig. 2:**
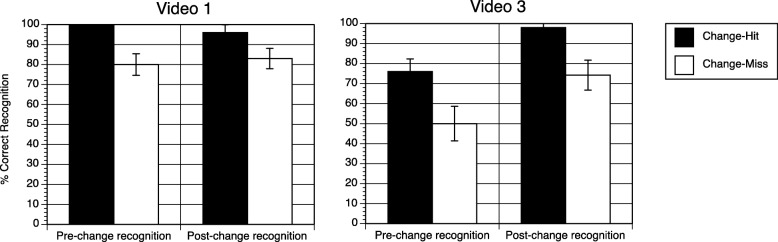
Percent accuracy on visual recognition for change-hit and change-miss trials in experiment 2. *Error bars* represent standard errors

Participants in experiment 2 detected 24% of the changes overall. However, change detection rates were very low in two of the videos (2.5% in video 2 and 5% in video 4) and more comparable to rates in experiment 1 for video 1 (31%) and video 3 (57%). Therefore, we tested for a link between change detection and recognition only in videos 1 and 3. Change detection was associated with increased recognition accuracy for pre-change properties in video 1 (pre-change, 100% recognition for change-hitters, 80% accuracy for missers, *p* = 0.0144 Fisher’s exact test; post-change 96% recognition for hitters and 84% for missers, X^2^ = 2.40, not significant) and for both pre- and post-change properties in video 3 (pre-change, 76% recognition for hitters and 50% for missers, X^2^(1) = 5.84, *p* < 0.025; post-change, 96% for hitters and 84% for missers, *p* = 0.002 Fisher’s exact test) (Fig. 2).

#### Gaze and change detection

Analyses testing whether gaze duration predicted change detection were also run on only videos 1 and 3. Change detections were entered into a generalized estimating equation analysis (again, all general estimating equation analyses in this section used video as a within-subject effect, assumed a binomial distribution, and an unstructured working correlation matrix structure) with pre-change and post-change region of interest gaze duration and video as predictors. The analysis demonstrated that gaze duration on neither the post-change (*X*^*2*^(1) = 3.159, *p* = 0.076) nor the pre-change ROI (*X2* < 1) predicted change detection. Table 1 lists pre- and post-change gaze durations for detected and undetected changes for each video.

Next, we tested whether gaze duration predicted recognition using data from all four videos. Recognition of pre- and post-change properties was entered into a generalized estimating equation with pre- and post-change region of interest gaze duration as covariates. The impact of gaze was nonsignificant for pre-change recognition (*p* > 0.37) and post-change recognition (*p* > 0.75). An analysis of gaze-to-recognition links for each individual video (using logistic regressions) suggested some links for video 3. For that video, gaze duration in the post-change ROI predicted pre-change recognition (*X*^*2*^(1) = 6.003, *p* = 0.014) and nonsignificantly predicted post-change recognition (*X*^*2*^(1) = 2.767, *p* = 0.10).

As in experiment 1, we also tested whether minimum gaze duration across pre- and post-change ROIs predicted change detection and observed this to be the case. For videos 1 and 3, a general estimating equation procedure demonstrated that detected changes (4.1 frames in ROI) were not associated with significantly higher minimum gaze duration than missed changes (3.9 frames; *X*^*2*^(1) = 5.391, *p* = 0.02). The frequency histogram of minimum gaze durations for change detection and misses for videos 1 and 3. Gaze durations were five frames or longer for 26% of missed changes, 10 frames or longer for 10% of missed changes and 30 frames or longer for no missed changes. For detected changes, gaze durations were five frames or longer for 34% of detected changes, 10 frames or longer for 13% of changes and 30 frames or longer for no detected changes. Similarly, for minimum longest within-ROI run-lengths, for 10% of missed changes gaze was in both regions for at least five continuous frames. For 1.1% of missed changes gaze was in both regions continuously for 10 frames. In comparison, for 9.8% of detected changes, gaze was in both regions for at least five frames. For 1.4% of detected changes gaze was in both regions continuously for 10 frames.

#### Change detection and learning

Participants got an average of 26%, 30%, 61%, and 39% correct on the pre-test for videos 1–4 (as scored by a rater out of three possible points for the single open-ended question for each video). The mean score on the post-test was 76%, 70%, 58%, and 50% correct for videos 1–4, respectively (compared with a nominal 25% guessing baseline for the four-alternative multiple choice questions used here). Regressions testing whether change detection and recognition predicted learning revealed no significant relationships. The basic regression predicting the mean content score on all questions across all videos included change detection, pre- and post-recognition, and pre-test scores (collapsed across all videos) was significant overall (*F*(3,76) = 4.986, *p* = 0.003). Pre-test scores predicted content scores (β = 0.388, *t* = 3.599, *p* < 0.001). Otherwise, neither change detection (β = − 0.025, *t* < 1) nor recognition accuracy (β = 0.079, *t* < 1) predicted content scores.

#### Descriptive analysis of gaze across ROI time bins for videos from experiments 1 and 2

**Fig. 3:**
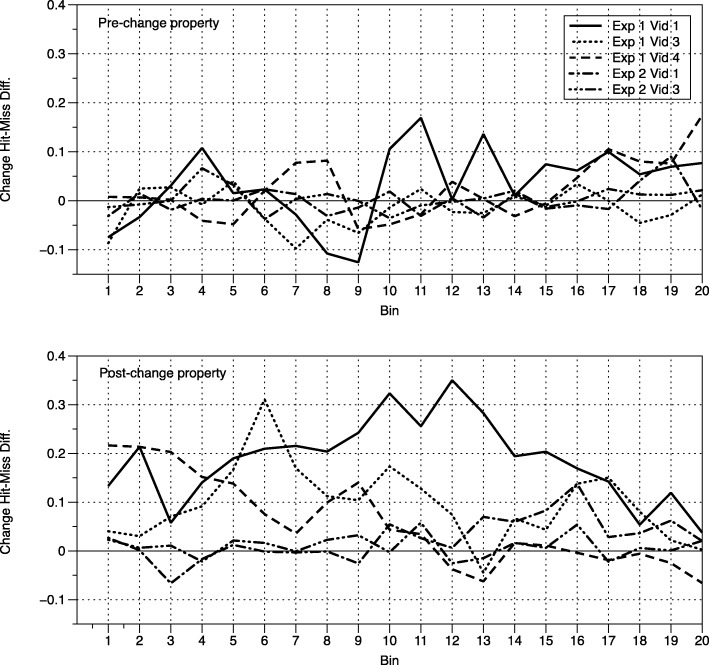
Difference in fixation probability between participants who did and did not detect the change, across time bins in pre- and post-change property regions of interest. Positive values indicate extent to which participants who detected the change looked at the property longer than participants who missed the change

To further assess the relationship between gaze and change detection, and between gaze and recognition, we tabulated proportion of time that gaze fell within regions of interest defined by pre- and post-change properties across each of 20 time bins scaled to the on-screen duration of pre- and post-change properties. We selected videos for which a substantial number of participants detected the change, eliminating videos 2 and 4 in experiment 2, and videos for which the pre- and post-change properties were visible for only a limited time (eliminating video 2 of experiment 1 for which the properties were visible for the entire video). For the remaining five videos, pre-change properties were on-screen for 4.03 to 6.07 s (so, each bin represents 202 ms to 303 ms) and post-change properties were on-screen for between 4.27 and 39.13 s (each bin represents 214 to 1950 ms). Figure 3 shows the extent to which participants who detected changes looked within the pre- and post-change property ROIs for a higher proportion of time than participants who did not detect the change. As the figure makes clear, participants who saw the change were more likely to look at the post-change property for a wide range of relative times throughout the on-screen availability of the property. Conversely, the weaker relationship between change detection and gaze within the pre-change ROI does not seem to have hidden time-bins that individually may have consistently differentiated participants who did and did not see the change.

## General discussion

The results of experiments 1 and 2 were consistent in revealing two key findings. First, there was again a clear relationship between change detection and recognition of the changing properties: participants were less accurate in recognizing the pre- and post-change properties when they missed changes, although recognition was, overall, reasonably accurate. Second, in both experiments, a substantial proportion of changes were missed even when gaze fell within both the pre- and post-change ROIs for time periods comparable to, or greater than, those used to argue previously in support of attentive blank stares (Caplovitz et al., 2008). In addition, we observed no relationships between change detection and overall learning, although this negative finding should be viewed with caution because in the absence of a no-video control group it is not clear how much participants learned overall.

Fundamentally, we take evidence that change blindness is associated with reduced visual recognition as evidence in support of the hypothesis that change blindness in this case is not an isolated failure to compare otherwise detailed and robust property representations, but rather reflects broader limits in the availability of visual representations of the changing objects. However, participants who missed changes were, overall, reasonably accurate at recognizing the changing properties (although it should be pointed out that above 50% recognition in this particular two-alternative test is not necessarily strong evidence of preserved representations because we did not counterbalance targets and distractors in the recognition tests). In addition, recognition failures cannot be considered conclusive evidence that changing properties have gone entirely unrepresented (Levin & Baker, 2015; Triesch, Ballard, Hayhoe, & Sullivan, 2003; Varakin, Levin, & Collins, 2007). Clearly, it is possible that some other test for the presence of representations of the changing features would reveal equivalent performance for participants who did, and did not, detect changes. For example, an implicit measure such as priming might reveal equivalent representations for changing properties whether or not participants detected the change. However, any such representational successes in the face of change blindness do not lessen the consequences of failures such as those we have observed: if participants cannot explicitly recognize a changing property, they cannot talk about it, draw upon it when they solve problems, or use it in deliberative learning.

Of course, one could argue that linking any lessening in recognition performance to change detection, especially when recognition is relatively accurate in the face of change blindness, might constitute a relatively low bar for demonstrating some kind of representational failure. As mentioned above, however, it is possible to observe equivalent and above-chance recognition in participants who do and do not detect changes (Angelone et al., 2003). So, if the specific failures documented in research using highly incidental tasks and/or in lab tasks can also be documented in more naturalistic and applied tasks where participants are focusing on material with the goal of learning, then the growing domain of representational failures may begin to encompass a substantial and important segment of visual experience, and this is one of the key goals of the current project.

In addition to the results on our recognition test, results from gaze and fixation patterns can help specify the patterns of attention associated with change detection in this case. First, these data demonstrated that there were quite a few instances where participants clearly fixated on both pre- and post-change properties and nonetheless failed to report the changes. As reviewed above, this finding has been reported previously (for example, O’Regan et al., 2000), but this is one of relatively few experiments where failure to detect changes in the face of pre- and post-change fixations has been documented in an applied setting where participants have both been warned that they will have to detect changes and are engaged in a task that naturally draws upon the visual structure encompassing the changing properties. The closest analog we know of is research by Vallières, Mallat, Tremblay, and Vachon (2015), who documented change blindness in the face of gaze in a simulated aircraft-monitoring setting. The key commonality to all of these settings is that they document situations where looking has provided ample opportunity to result in representations sufficient to detect changes. This is consistent with a range of findings that demonstrate failures to reduce change blindness in the face of other representation-creation opportunities such as scene previews (for example, Rensink, O’Regan, & Clark, 2000).

Other patterns of results in the eye movement data may provide useful evidence about gaze patterns that may signal representation-creation and comparison. In particular, we observed that gaze duration to pre-change properties generally did not predict change detection while change-hitters and change-missers were sometimes differentiated by gaze duration in the post-change properties. This is consistent with the hypothesis that variations in the amount of attention given to an object do not map directly on to variations in the prevalence of representations that are by default used prospectively to detect changes. The link between post-gaze and change detection was sometimes significant, but this could have occurred because change detection caused increased inspection of the post-change ROI, not because gaze in the ROI increased change detection. However, the pattern of differentiation of gaze between change-hitters and change-missers across the time course of the post-change ROI is potentially interesting because the gaze advantage for change-hitters is not specific to the onset of the post-change object, which one might expect if variation in the strength of a stimulus-driven onset induced change detection.

Findings such as these may also support useful conclusions about the application of research on change blindness. Most concretely, these findings make clear that change blindness is easily observed in graphical user interfaces, and a look at Additional file 1 gives a good sense of the magnitude of changes that can go undetected. In addition, we observed that change detection and visual recognition were not associated with learning of the specific contents in the videos. Of course, this is a negative finding, so it should not be considered strong evidence that the two are generally unassociated. However, the importance of reporting negative findings is often discussed (see, for example, Matosin, Frank, Engel, Lum, & Newell, 2014; Oldehinkel, 2018), and we have observed change detection-learning links in other settings. In a recent on-line experiment we tested learning and change detection in brief 45-s outtakes from the videos used in these experiments and found that increased change detection was associated with increased learning. Further research might, therefore, test the hypothesis these briefer lessons are less conceptually organized and therefore involve visual encoding that more closely links attention, representation, and learning, while longer more conceptually organized videos lessen these links. Such research could both draw upon and inform human–computer interaction design principles that attempt to balance the need to be aware of changes with the need to contextualize and elaborate upon visual infromation (St. John & Smallman, 2008).

Also, more visual forms of learning might be associated with the sorts of representations that support change detection and visual recognition. It would be particularly interesting to systematically test for representations that represent successive generalizations away from visual recognition of changing properties. For example, Varakin et al. (2007) found that change detection was associated with recognition of the changing properties, but not for a non-changing property on the same object. However, this was an entirely incidental change detection experiment in which participants did not expect to detect changes or to learn anything, which might tend to limit the impact of attending to an object and seeing it change.

## Conclusions

So, what have we learned about change blindness during screen-captured video learning? First, change blindness is a relatively frequent occurrence, even in situations where the student is motivated to attend to visual information and knows that he/she will need to be on the lookout for changes. Second, failures to detect changes in this setting are not completely isolated—they are associated at least with relative failures to represent visual properties. In addition, these failures have occurred even where eye tracking has provided evidence that visual information necessary to detect the change has fallen within the participants’ gaze. That said, the situation is not entirely hopeless. Participants recognized the changing properties reasonably accurately, and they have leaned just as much content as students who detected the change. This implies that visual processing in this setting is at least partially independent of content learning. Future research might ask how failures of visual awareness such as change blindness might be linked with variations in more concrete visual learning that could be expected to accumulate with experience, especially in settings characterized by relative stability of visual configurations (such as dialog boxes and windows). For example, it is possible that some settings cause relatively tight integration of attention and representation resulting in good visual learning by default, while other situations might be characterized by much less integration and learning that requires much more explicit focus of attention and conceptual elaboration.

## Additional file

Additional file 1: Illustrations and descriptions of changes in experiments 1 and 2. (DOCX 3462 kb)

## Abbreviation

ROI: Region of interest
