# Supplementary material for: Are failures to look, to represent, or to learn associated with change blindness during screen-capture video learning?
Source: Cogn Res Princ Implic. 2018 Dec 27;3:49. doi: 10.1186/s41235-018-0142-3 (PMC6306372; doi:10.1186/s41235-018-0142-3)
Supplement: Supplementary file 1 — Illustrations and descriptions of changes in experiments 1 and 2. (DOCX 3462 kb) [file 41235_2018_142_MOESM1_ESM.docx]

Additional file 1: Illustrations and descriptions of changes in Experiments 1 and 2.

**Experiment 1**

Video 1


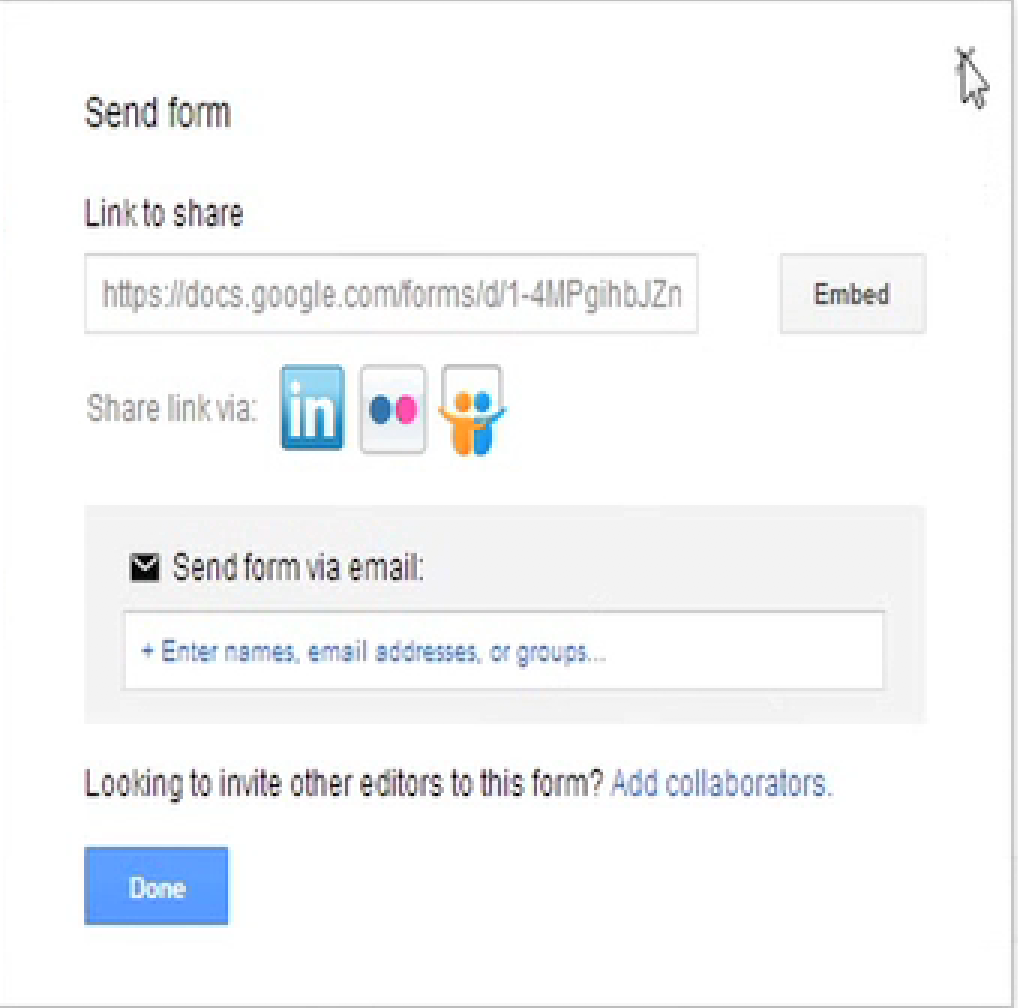

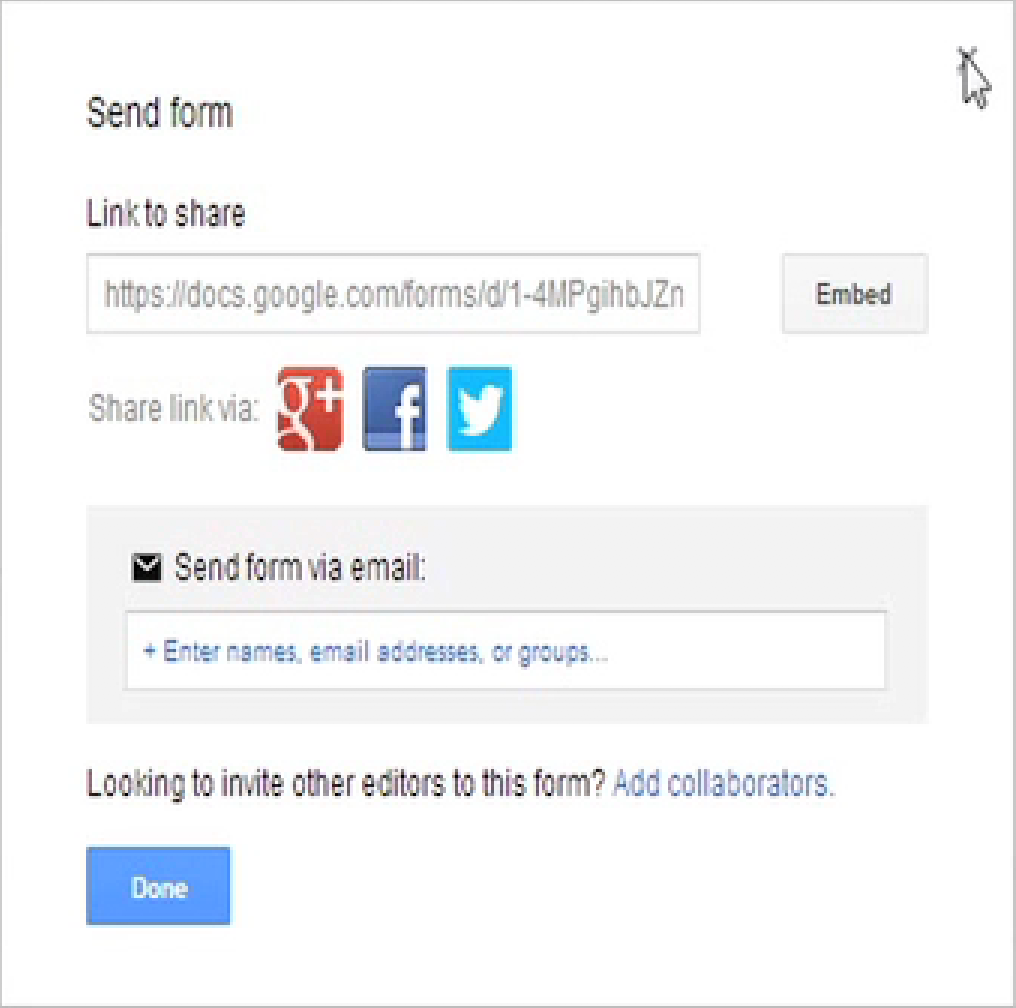


16.7% change detection. Pop-up dialog appeared at 3:09 in the center of the screen. Visible 4.0 sec pre-change and 5.5 seconds post-change when the pop up screen was closed. Pixel area of change=2,255.90

Video 2


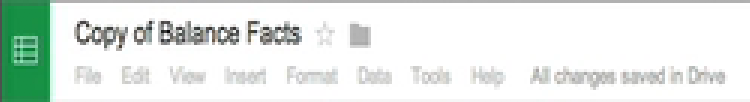

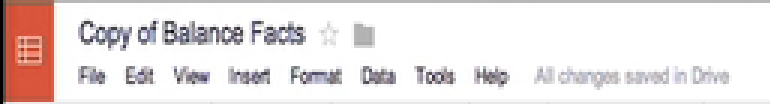


56.4% change detection. ROI was visible the entire length of the video. Visible 1:10 pre change and 4:09 post change. Pixel area of change=577.76

Video 3


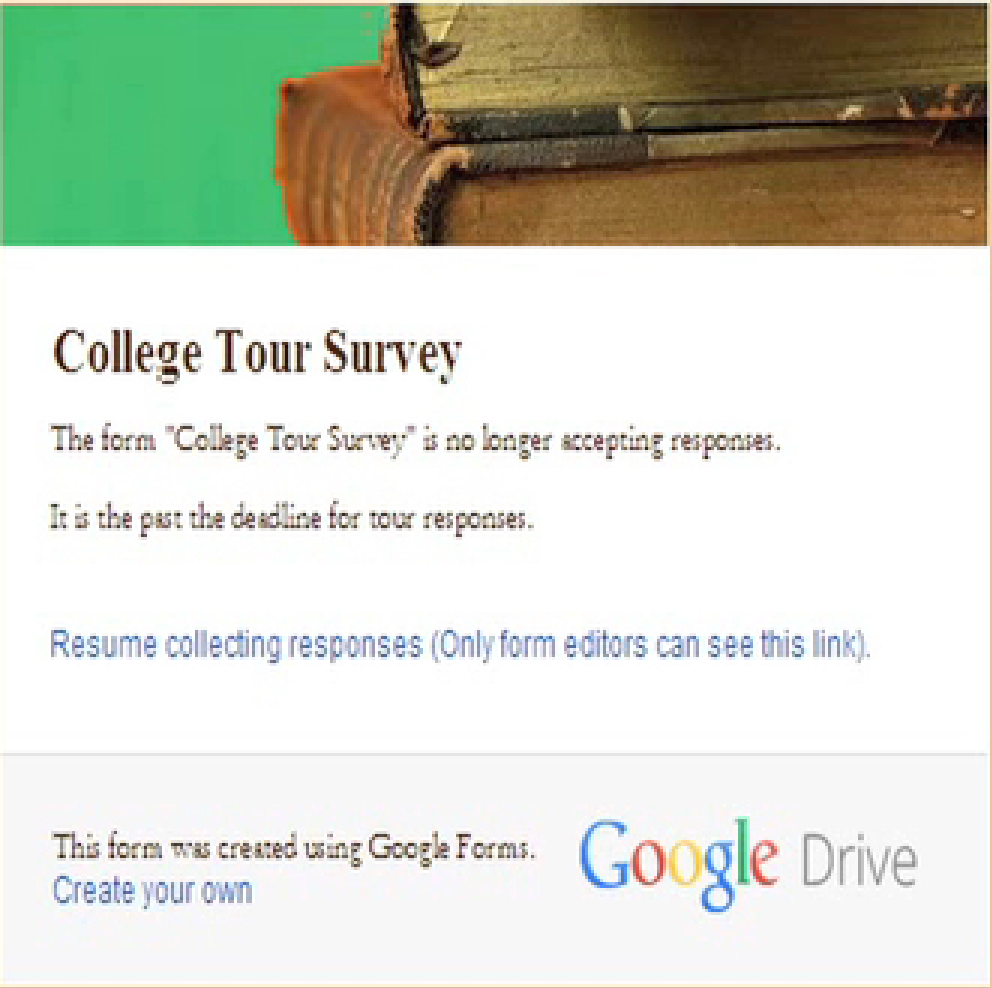

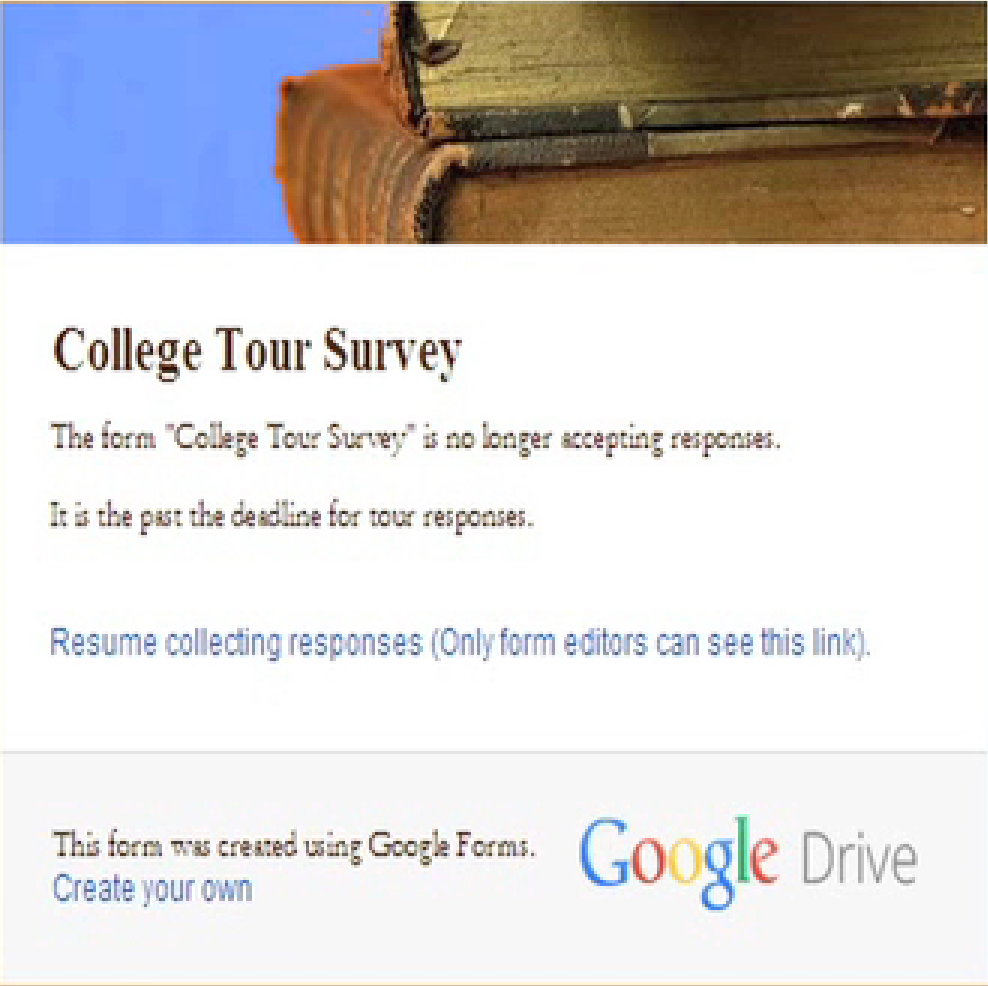


52.5% change detection. Pop-up dialog appeared at 4:08 in the center of the screen. Visible 3.7 sec pre-change and 7.0 seconds post-change when the pop up screen was closed. Pixel area of change=10,240.0

Video 4


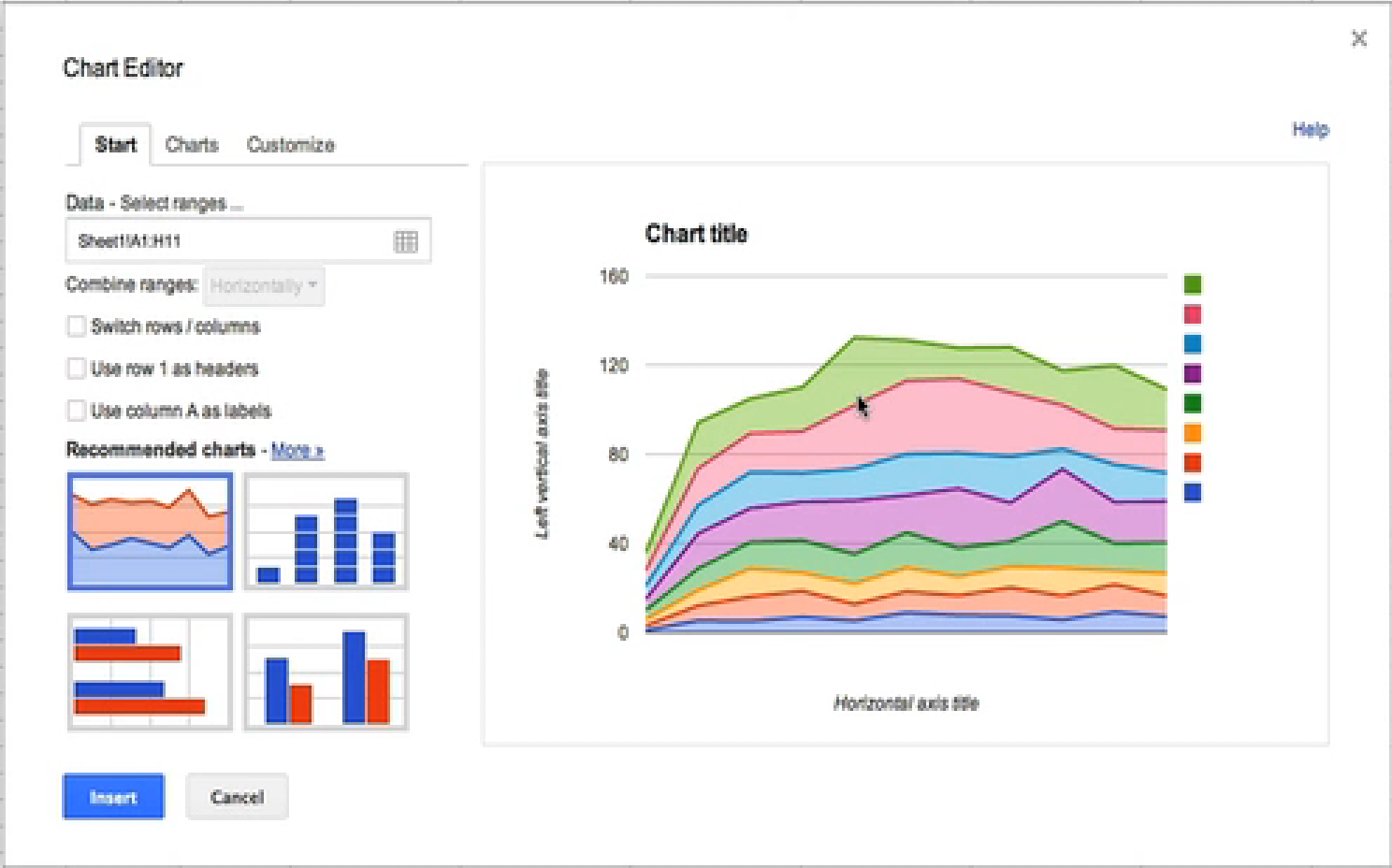

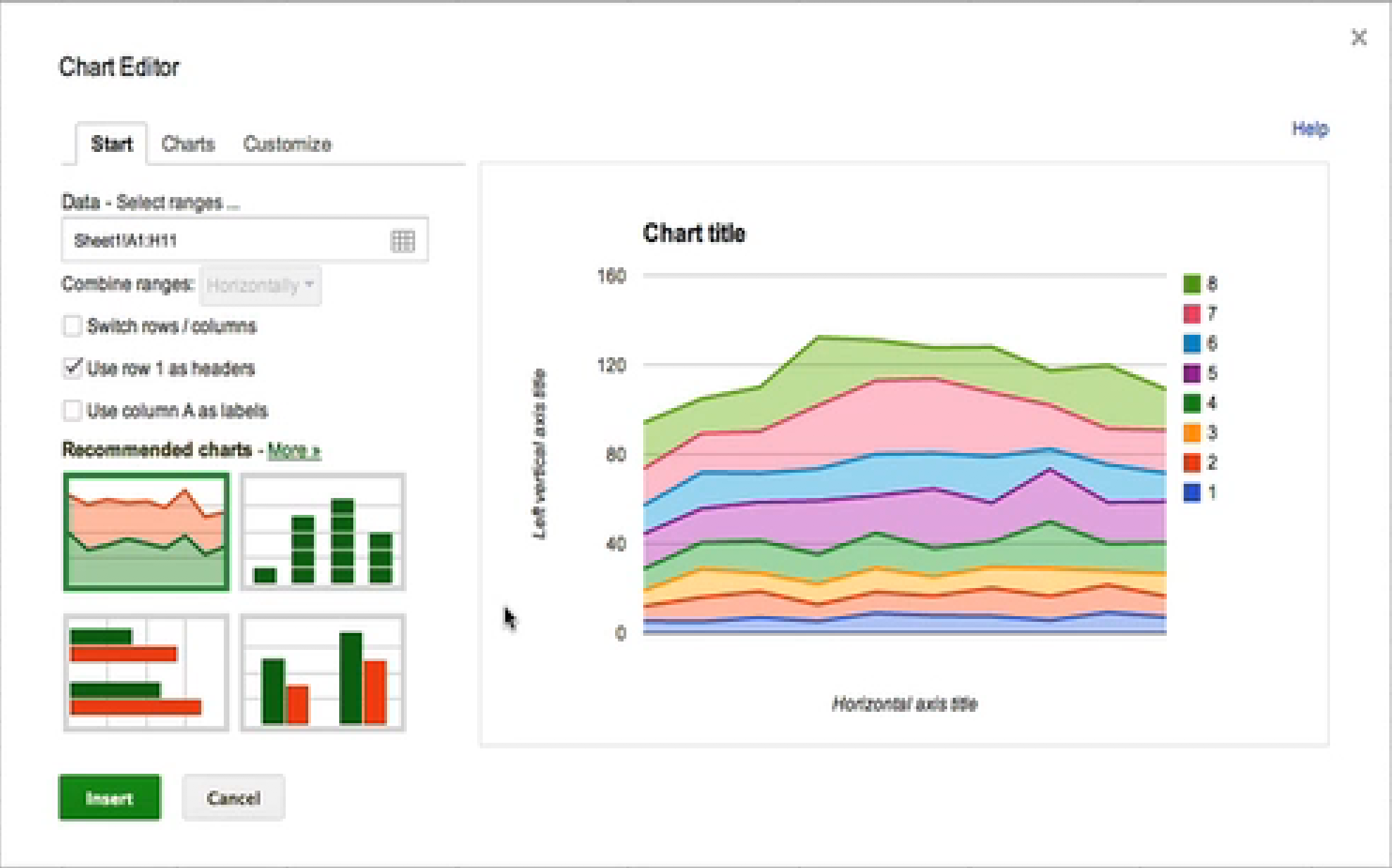


64.1% change detection. Pop-up dialog appeared at 57.6 sec in the center of the screen. Visible 6.1 sec pre-change and 39.1 seconds post-change when the pop up screen was closed. Pixel area of change=11,962.0

**Experiment 2**

Video 1


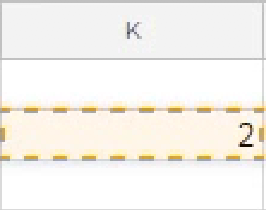

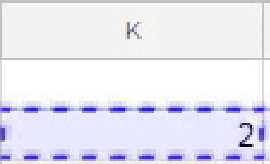


31.2% change detection. The cell was highlighted at 8:02. Visible 6.4 sec pre-change and 6.7 seconds post-change when the cell was no longer highlighted. Pixel area of change=2,342.91

Video 2


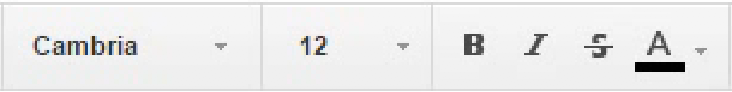


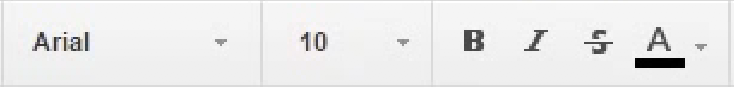


2.5% change detection. ROI was visible the entire length of the video. Visible 2:21 pre change and 2:46 post change. Pixel area of change=6,120.85

Video 3


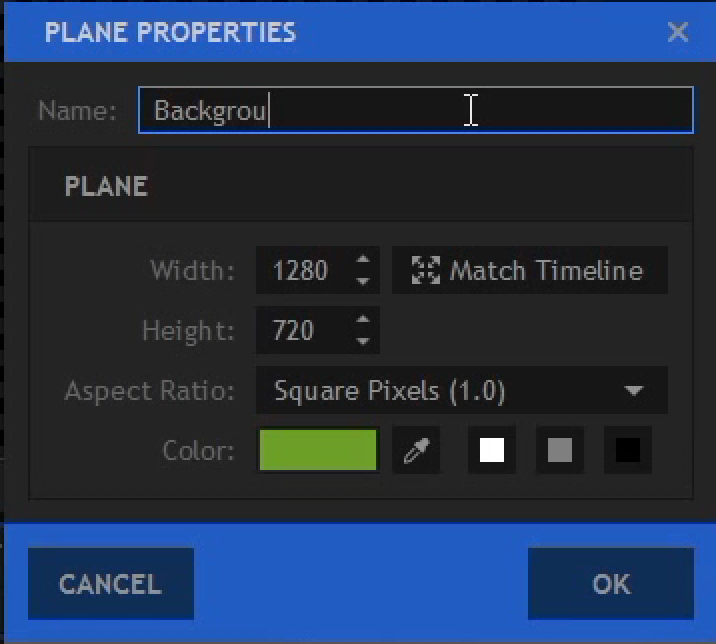

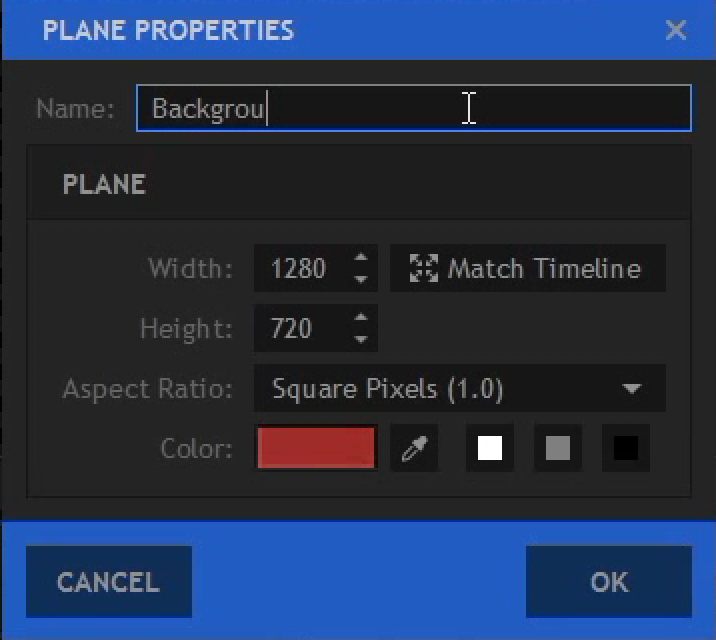


57.5% change detection. Pop-up dialog appeared at 1:06 in the center of the screen. Visible 4.93 sec pre-change and 4.7 seconds post-change when the pop up screen was closed. Pixel area of change=1,226.22

Video 4


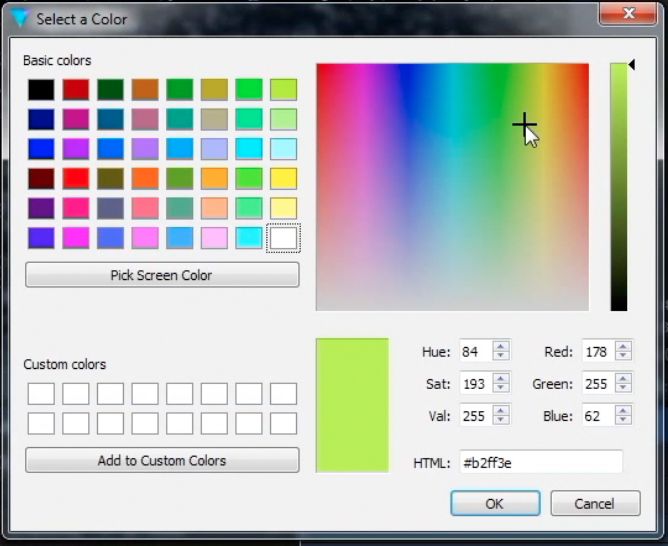

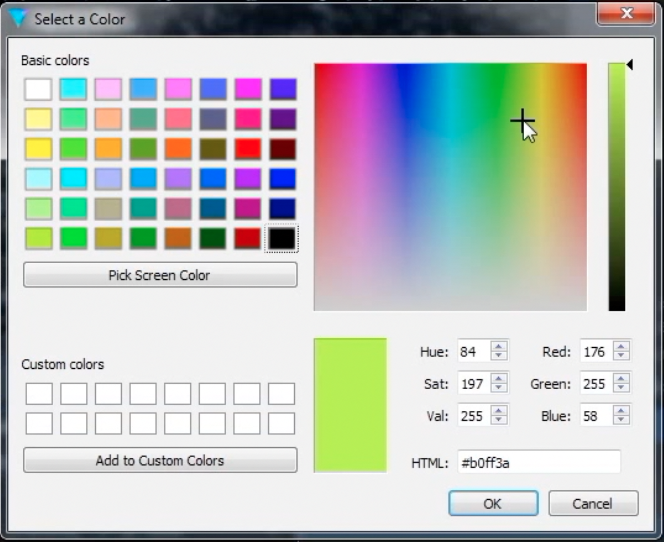


5.0% change detection. Pop-up dialog appeared at 29.96 sec in the center of the screen. Visible 4.9 sec pre-change and 4.2 seconds post-change when the pop up screen was closed. Pixel area of change=32,320.49
